# History-dependent ion transport through conical nanopipettes and the implications in energy conversion dynamics at nanoscale interfaces†
†Electronic supplementary information (ESI) available. See DOI: 10.1039/c4sc02195a



**DOI:** 10.1039/c4sc02195a

**Published:** 2014-08-20

**Authors:** Yan Li, Dengchao Wang, Maksim M. Kvetny, Warren Brown, Juan Liu, Gangli Wang

**Affiliations:** a Department of Chemistry , Georgia State University , P.O. Box 3965, 50 Decatur St. SE , Atlanta , Georgia 30303 , USA . Email: gwang@gsu.edu ; Tel: +1-404-413-5507

## Abstract

The dynamics of ion transport at nanostructured substrate–solution interfaces play vital roles in high-density energy conversion, stochastic chemical sensing and biosensing, membrane separation, nanofluidics and fundamental nanoelectrochemistry. Further advancements in these applications require a fundamental understanding of ion transport at nanoscale interfaces. The understanding of the dynamic or transient transport, and the key physical process involved, is limited, which contrasts sharply with widely studied steady-state ion transport features at atomic and nanometer scale interfaces. Here we report striking time-dependent ion transport characteristics at nanoscale interfaces in current–potential (*I*–*V*) measurements and theoretical analyses. First, a unique non-zero *I*–*V* cross-point and pinched *I*–*V* curves are established as signatures to characterize the dynamics of ion transport through individual conical nanopipettes. Second, ion transport against a concentration gradient is regulated by applied and surface electrical fields. The concept of ion pumping or separation is demonstrated *via* the selective ion transport against concentration gradients through individual nanopipettes. Third, this dynamic ion transport process under a predefined salinity gradient is discussed in the context of nanoscale energy conversion in supercapacitor type charging–discharging, as well as chemical and electrical energy conversion. The analysis of the emerging current–potential features establishes the urgently needed physical foundation for energy conversion employing ordered nanostructures. The elucidated mechanism and established methodology can be generalized into broadly-defined nanoporous materials and devices for improved energy, separation and sensing applications.

## Abstract 2

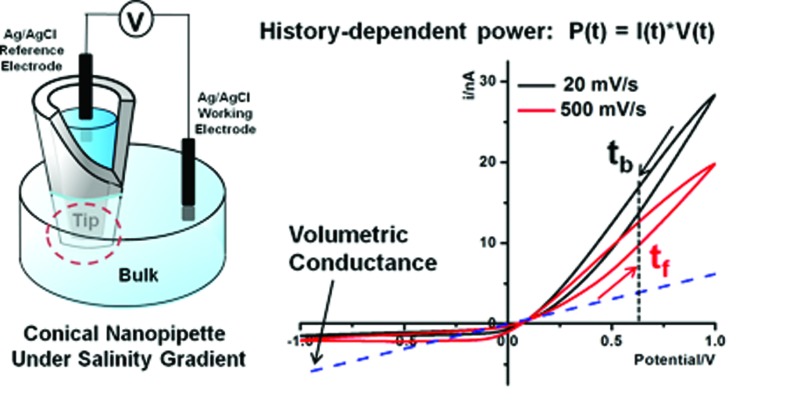
We report striking time-dependent ion transport characteristics at nanoscale interfaces in current–potential (*I*–*V*) measurements and theoretical analyses.

## Introduction

Charge transport at solid–solution interfaces is a key step in fundamental electrochemistry,^1,2^ energy technology (*i.e.* charging and discharging of supercapacitors, fuel cells and batteries),^3–5^ separation (*i.e.* desalination, filtration),^6^ sensing (*i.e.* DNA sequencing and stochastic single molecule detection),^7–9^ and natural processes in biology (*i.e.* ion channels and pumps) and geoscience.^10^ Materials and devices with defined atomic and nanometer scale pore structures have great potential to enhance the efficacy and/or efficiency of these applications.^11,12^ At nanoscale interfaces, novel transport phenomena emerge that require further experimental and theoretical studies. Representative features include steady-state ion current rectification (ICR),^13–15^ apparent inductive behavior,^16^ memcapacitance,^17^ and ‘abnormal’ hysteresis and capacitance responses under high-frequency electric field stimulation^18–20^ observed in different channel-type nanodevices.

The ion transport process is associated with energy conversion and characterized by current (*I*), potential (*V*) or power (*I* × *V*). Recent advances in steady-state studies pave the way for a better understanding of the dynamic ion transport at nanoscale interfaces, which is urgently needed to advance three types of important applications. First, ion transport through porous electrodes is the physical and rate-limiting step in the charging and discharging of electrochemical capacitors (supercapacitors). Supercapacitors are widely used as energy devices complementing or competing with batteries, *etc.* One of the key merits of supercapacitors is that they deliver high power. This high power unfortunately leads to a major limitation: they don't last long before a recharge is needed. It is therefore highly desirable to control their power output, or charging and discharging kinetics. Two other applications are inspired by the natural processes of protein ion pumps in which chemical energy (*i.e.* ATP–ADP) is converted into electrochemical potential in terms of action potential, concentration gradient, *etc.* In the second type of applications, electrical energy is harvested from the salinity gradient, the pressure driven flow, *etc.* with classic ion selective membranes and, recently, nanopores. Third, selective ion transport will enable ion enrichment/depletion or separation (*i.e.* desalination by the consumption of electricity, light, *etc.*).^21–29^ The main limiting factors for improvement reside in the lack of fundamental physical understanding of the transport dynamics and the need for significant enhancements in methodology.

**Fig. 1:**
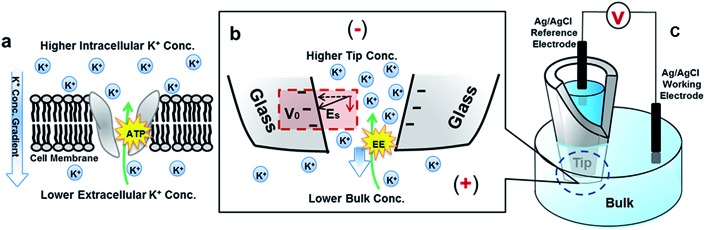
Scheme of a nanopore in a transmembrane protein and solid-state nanopipette under an ion concentration gradient. (a) A K^+^ ion pump in the lipid bilayer that transports K^+^ against the concentration gradient by ATP hydrolysis. (b) Enlarged view of the nanopipette tip under a high-tip-low-bulk concentration gradient. Quartz/glass serves as the counterpart of the insulating lipid bilayer. K^+^ ions could migrate against the concentration gradient driven by external electrical energy (EE). Cl^–^ ions are omitted due to their lower contribution to the ion flux as a result of repulsion from negative surface charges. The surface potential (*V*_0_) and direction of the surface electric field (*E*_s_) are highlighted. (c) Experimental setup. The bias is defined as bulk *versus* tip.

Our approach to analyze dynamic transport through solid-state nanopores in comparison with protein ion pumps is illustrated in Fig. 1. A nanopore in a quartz substrate allows the exchange of ions between the tip and bulk solutions. Unlike the energy from ATP hydrolysis being converted into a concentration gradient, an alternating bias is applied to drive the dynamic ion transport. Time-dependent *I*–*V* studies reveal dynamic transport features inaccessible in the commonly-adopted steady-state analysis. For clarification, a comparison between steady-state and dynamic/transient transport is analogous to that of uniform velocity *versus* acceleration/deceleration. The dynamic ion transport benefits from substantial contributions from the tip-localized surface electric field. Furthermore, the salinity gradient on the two sides of the nanopore in the bulk solution is introduced. The predefined salinity gradient enables the analysis of the energy conversion between the electric and chemical potentials. The conversion efficiency is time-dependent and characterized by power. The energy conversion efficiency or power is found to depend on the previous conductivity state. This history-dependent feature, not captured in previous studies, enables the elucidation of drastic enhancement by the surface electric field.

As highlighted in Fig. 1(b), the surface electric field has a component *E*_s_ along the direction of the ion flux (or applied field) that distinguishes this asymmetric nanogeometric platform from symmetric nanochannels, and from amorphous pores in ensemble membranes. The transport-limiting tip region has an orifice radius of a few tens of nanometers. Correspondingly, high efficiency energy conversion is achieved in terms of the nanointerface enhanced ion transport.

We start by introducing time-dependent hysteresis *I*–*V* features in symmetric tip–bulk concentration conditions. A unique non-zero *I*–*V* cross-point and pinched *I*–*V* loops are then employed as characteristics to evaluate the efficacy of the energy conversion and pumping ions against the concentration gradient in asymmetric concentrations. Theoretical equations are developed by correlating the cross-point potential and the current clamping in *I*–*V* and *V*–*t* studies, respectively, through which significant surface impacts and the contributions by diffusion and migration to the overall measured current signal are elucidated. The findings can be generalized for different types of substrate–solution interfaces, therefore broadly impacting the aforementioned applications.

## Results and discussion

### Dynamic *I*–*V* features from ion transport through a quartz nanopipette under symmetric tip–bulk concentrations

**Fig. 2:**
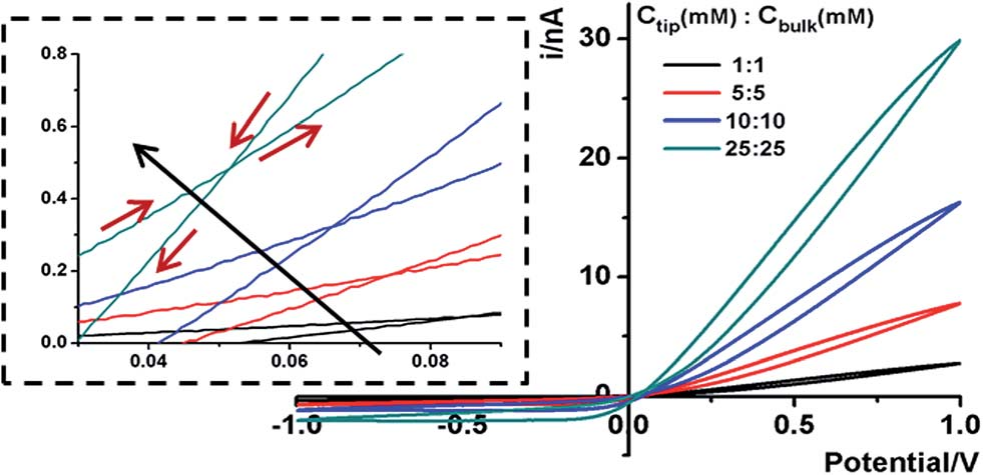
Representative *I*–*V* features of a 60 nm radius nanopipette in symmetric tip–bulk KCl concentrations. The scan rate is 100 mV s^–1^. The inset shows the scan directions and the cross-point positions of all *I*–*V* curves. The shift in cross-point with the increase of concentration is indicated by the long arrow.

Representative ionic current responses of the conical nanopipettes under a scanning triangular potential waveform are shown in Fig. 2. The nanogeometry is analyzed by scanning electron microscopy and conductivity measurements (Fig. S1†). The immediately notable feature in each curve is that in the cyclic scans (the scan directions are shown in the inset), two hysteresis loops are separated by a unique cross-point at a small positive bias rather than at the origin (0,0). These large hysteresis effects at such a low frequency range indicate significant surface effects on the pulled conical nanopipettes. Similar *I*–*V* features on a new device platform are in agreement with our recent report on pinched hysteresis loops and a non-zero cross-point in the ion transport through bench-top fabricated conical nanopores, and thus strongly support the proposed analysis therein.^20^ Such dynamic *I*–*V* features have not been observed in steady-state transport studies using broadly defined channel-type nanodevices to the best of our knowledge. For comparison with steady-state responses, the non-linear *I*–*V* curve of each scan segment, specifically with the current amplitude being higher at one bias than that at the opposite bias polarity, corresponds to the well-known ICR behavior.^15,30–34^ A key difference is that the *I*–*V* curve does not necessarily cross through the origin (0,0), as approximated in previous reports.

The intriguing observation of a unique non-zero *I*–*V* cross-point separating opposite hysteresis loops reveals unknown fundamental ion transport dynamics at nanoscale interfaces. For *I*–*V* measurements in solution, a hysteresis loop is normally interpreted as capacitive responses. On the low conductivity side (defined as a more negative bias than the cross-point), the ionic current displays a hysteresis corresponding to normal capacitive behavior, or a positive phase shift of current responses with respect to the applied scanning potential waveform. On the high conductivity side, however, the measured current shows a hysteresis corresponding to a negative phase shift, or negative capacitive responses. This negative phase shift is in agreement with the ‘apparent inductive’ or ‘negative capacitance’ behaviors reported in impedance experiments and molecular dynamic simulations on different types of nanochannel platforms respectively.^16,17^ Based on steady-state ICR studies, these dynamic ion transport features observed from different nanochannel platforms have been attributed to surface electric field effects.^16–20^

The ionic strength of the solution is systematically varied to reveal its impact on the hysteresis *I*–*V* features by the surface electric field shown in Fig. 2. On an SiO_2_ substrate, the surface electric field originates from the negative surface charges due to the deprotonation of surface silanol groups. With an increase in KCl concentration, electrostatic interactions between mobile ions and surface charges are more effectively screened. The cross-point potential (*V*_cpp_) decreases accordingly. The current (in general and at the cross-point) increases at higher KCl concentrations because more charge carriers (mobile ions) are accessible to the current-limiting nanotip region.

The cross-point can be understood as corresponding to the applied field that balances the effective surface electric field (*E*_s_) across the quartz membrane at the nanotip region in potential scanning experiments (Fig. 1b), as explained in our early reports.^20,35^ For clarity, we use the elongation/compression of a spring as an analogy here. The spring mimics the nanopipette, with the relaxed state (length *L*) corresponding to the intrinsic surface charge/potential and nanogeometry. Next we consider the surface effects in the ionic solution as analogous to the weight added to the spring. The spring will elongate differently with different weights (*L* + *W*). This scenario mimics the cross-point that appears at a non-zero position and depends on the ionic strength of the solution. Without an external bias, the ion distribution at the tip region of the nanopipette is established by the intrinsic surface electric field. The EDL at the nanointerface is at rest or unpolarized. The net ion flux or transport current is zero. Under an external bias, the ion flux will alter the ion distribution, or polarize the EDL structure at the nanotip region. Accordingly, surface effects emerge and affect the measured current because the surface electric field is no longer balanced. The true steady-state, with non-zero flux/current, is established when the applied bias is equal to the effective surface potential across the nanotip.

If the spring setup is further stretched by an external force to *L* + *W* + *P*, (*P* represents the impact of an external bias in *I*–*V* measurements), regardless of further stretching or release by the external force, the spring setup itself will tend to recover toward *L* + *W*. Similarly, regardless of whether the bias is scanned in the forward or backward direction, *i.e.* from +0.5 V away from or toward the cross-point, within one of the hysteresis loops, the surface EDL tends to recover to the rest state. In other words, surface effects will continue to enrich the ions in the high conductivity loop and deplete the ions in the low conductivity loop regardless of the bias scan direction. Furthermore, because the ion flux is not zero immediately before the bias is scanned to zero, non-zero current signals are detected and ‘memorize’ the previous conductivity states due to the still-polarized EDL. The true steady-state is established at the cross-point when the extent of EDL polarization leads to a balance in the magnitude of the surface and applied electric fields along the ion transport direction.

Steady-state ICR (*I*–*V* branch from the backward scan is a better mimic due to longer accumulation) results from the overlapping effect of the intrinsic surface electric field with respect to a constant applied electric field. Accordingly, the hysteresis effects are attributed to the differences in the kinetics of the applied potential (determined by the scan rate or frequency) with respect to the corresponding ion transport through the nanopipette. It is worth pointing out that the direction of *E*_s_ is solely determined by the surface charge polarity, albeit its magnitude varies at different ionic strengths and applied biases because it depends on the ionic distribution at the nanotip. Therefore, at a bias more positive than the cross-point, the surface field consistently facilitates ion transport driven by the applied field regardless of the respective magnitude, thereby reducing resistance and causing negative capacitance, corresponding to the high conductivity states and negative *I*–*V* hysteresis.

This non-zero cross-point is employed as a signature in the following discussions because it is stable and unique for each measurement system (Fig. S2–S4†). At each scan rate, the *I*–*V* curves from multiple scans overlap with the variation in cross-point within 5 mV (the first or first few segment(s) are discarded to better present stable *I*–*V* responses). This variation is acceptable within experimental errors, such as imperfect Ag/AgCl electrode preparation, thermo agitation and external interference, *etc.* The *I*–*V* features are also found to be independent of the initial potential or initial scan direction. A small variation in the cross-point potential could be observed at less than 10 mV at the different scan rates employed in this report. This variation intensified at higher scan rates, which has been attributed to the charging and discharging of the quartz substrate (through exterior interfaces).^35^ The effect is ignored in the following discussion because it is insignificant with regards to the trend discussed in this report.

### Dynamic ion transport through a quartz nanopipette under asymmetric tip–bulk concentrations

**Fig. 3:**
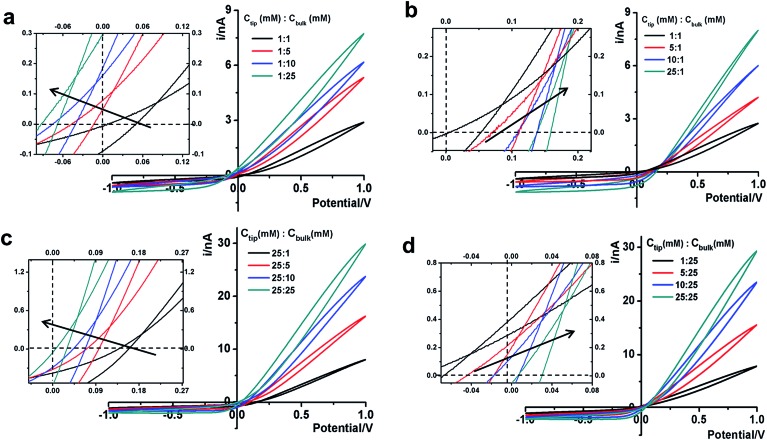
Trends of the *I*–*V* features in asymmetric tip–bulk KCl concentrations. The *I*–*V* curves of the 60 nm nanopipette in the following *C*_tip_–*C*_bulk_ concentration combinations: (a) common low tip conc. (b) Common low bulk conc. (c) Common high tip conc. (d) Common high bulk conc. The scan rate is 100 mV s^–1^. The inset shows the shift in the cross-point w.r.t. the origin (0,0). Each curve was collected after multiple scans until the last five repeated scans overlapped, confirming that a stable concentration gradient was established.

Next we analyze the dynamic ion transport under a concentration gradient across the nanopore driven by an external bias. A series of *I*–*V* curves using a single nanopipette in asymmetric tip–bulk KCl concentrations are presented in Fig. 3. The feature near the cross-points can be seen in the insets. Similar trends were obtained from different nanopipettes (Fig. S5†). Systematic studies using the same nanopipette eliminate the impact of possible imperfections in the nanodevice geometry, particularly the transport-limiting nanotip interior region that could not be directly characterized. Because the nanopipette geometry remains constant in these measurements, geometric effects can be avoided. This approach enables a direct correlation of the transport features with surface charge effects, which has been a long standing challenge due to the heterogeneous nature of the surface charge distribution, particularly at nanoscale interfaces. As a reminder, surface effects are known to be significant and even determinant factors in the ion transport processes through various channel-type nanodevices.

**Fig. 4:**
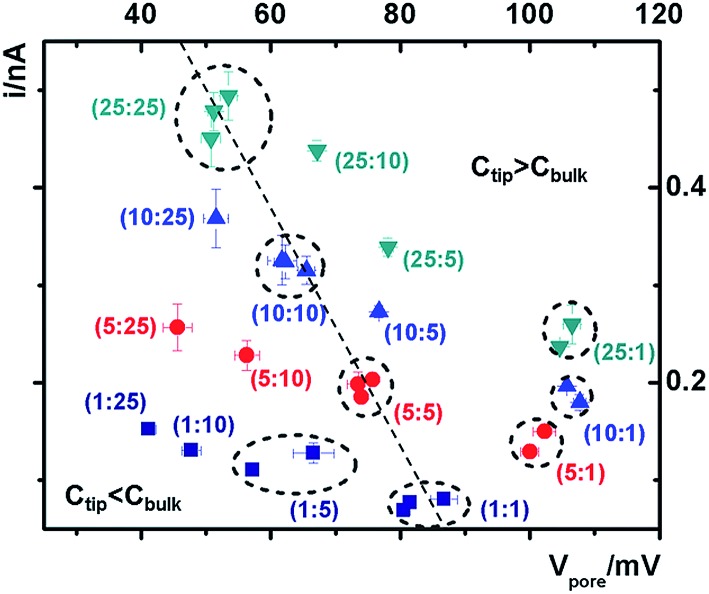
Analysis of the potential-corrected cross-points under various tip–bulk concentration gradient combinations. The error bars on each point show the standard deviations from five repeated scans under each condition. Between each asymmetric concentration measurement, symmetric ones with the same tip concentration were measured to validate the efficacy of loading and replacing the solutions, particularly inside the tip. Data from repeated measurements in the same concentration combination (both symmetric and representative asymmetric) are circled to highlight the reproducibility of the measurements.

The cross-points of these systematic concentration combinations are summarized in Fig. 4. Because the two Ag/AgCl wires are soaked in different KCl concentrations, unlike those in the same KCl solution, the potential drops at the two electrode–solution interfaces do not cancel each other and need to be corrected. The net redox potential (*V*_redox_) is described by the Nernst expression. The total bias at the cross-point (*V*_CPP(*I*–*V*)_) therefore includes *V*_redox_ and *V*_pore_ (the potential drop across the nanopore), as expressed in eqn (1). *V*_pore_ includes both the surface field and the concentration gradient effects discussed next.1
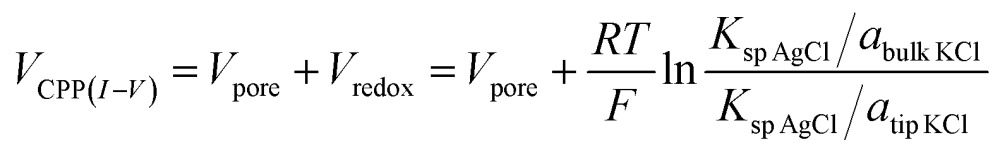
in which the activity of Ag^+^ (*a*_Ag^+^_) is approximated as concentration, which is inversely proportional to that of Cl^–^. *R* is the gas constant, *T* is temperature and *F* is Faraday's constant.

At each specific tip concentration, an increase in the bulk concentration will cause *V*_pore_ (or the measured *V*_cpp_) to shift toward a more negative bias. At each specific bulk concentration, an increase in the tip concentration will cause a positive-shift of *V*_pore_. The data also suggest that comparable concentration gradients induce similar magnitudes of shift of *V*_cpp_. At even higher concentration gradients, the cross-points could deviate from the trend presented in Fig. 4 (shown in Fig. S6a & b†). The deviation might be associated with the challenge to establish a stable and controllable concentration gradient, which is a prerequisite for this report.

With an increase of the bulk or tip KCl concentrations, the current increases due to the increase in accessible charge carriers on either side of the transport-limiting nanotip region. The cross-point current is more sensitive to the tip concentration and to a lesser extent the bulk concentration. This is because the measured current is primarily limited by a segment inside the nanopipette orifice. The current data at ±1 V of each curve are listed in Table S1.† Further current analysis requires surface charge parameters at the nanotip that are known to be heterogeneous and thus unavailable for individual nanodevices. This is currently being addressed in a combined experimental and simulation study.

### Balance of diffusion and migration flux by zero current clamping

Under the low potential amplitude and other experimental conditions employed here, ion flux arises primarily from (1) diffusion determined by the concentration gradient and (2) migration regulated by both the applied and surface electric fields.^31,32,36^ The measured current signals reflect the net ion flux limited by the nanotip region. Chronopotentiometry is employed next to elucidate the respective ion flux contributions by migration and diffusion. The applied potential is recorded over time until it reaches a stable value *V*_rev(*I*–*t*)_, during which the measured current is clamped to zero. This corresponds to no net flux of charges through the pore. In other words, at zero current, the ion flux driven by the external bias balances that driven by the concentration gradient. As expected in symmetric tip–bulk concentration conditions, the diffusion potential (*V*_diff_) is zero (within the experimental error of a few millivolts). Analogous to the cross-point potential analysis in *I*–*V* studies, the reversal potential (*V*_rev_) includes *V*_redox_ in addition to the diffusion potential, as expressed in eqn (2).2
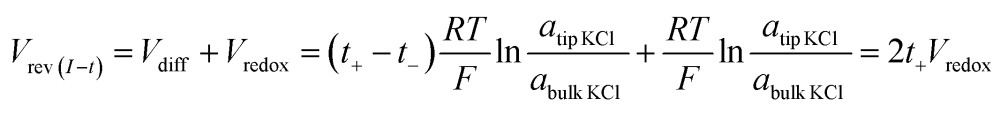



**Fig. 5:**
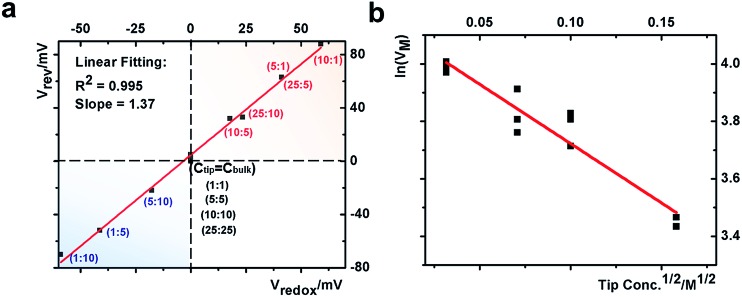
(a) Correlation between the measured reversal potential and the redox potential. (b) Correlation between the corrected transmembrane potential with the square root of the electrolyte concentration.

This quantitative correlation is affirmed by the linear fitting of the measured *V*_rev_ *versus* theoretical *V*_redox_ shown in Fig. 5a. The *V*_rev_ values and representative *V*–*t* curves are included in Fig. S7 and Table S2.† From the slope, the cation transference number is determined to be 0.69 for this nanopipette. Since ion selectivity is a significant parameter in membranes for desalinization, and broadly defined separation science and energy technologies, it is exciting for this analysis to directly characterize the cation selectivity of a single nanopore experimentally. Because the K^+^ and Cl^–^ mobilities are comparable, no diffusion potential is expected in the bulk solution (the transference numbers are 0.5 each). The large cation transference number reflects the surface electric field effect, which induces asymmetric cation and anion diffusional flux and makes this nanodevice cation-selective. The correlation in eqn (2) is further validated by the results from the different nanopipettes analyzed in Fig. S8.† The variation in these *t*_+_ values suggests heterogeneous surface charge effects of the individual nanopipettes.

In our earlier studies based on a conical nanopore platform in symmetric concentrations, the effective transmembrane potential (*V*_M_) was found to depend on the electrolyte concentrations by a square root function. In the expression shown in the last portion of eqn (3), analogous to the Debye length description in classic double layer theory, *V*_0_, *A* and *V*_e_ correspond to the surface potential inside the nanotip, a constant that is temperature dependent, and non-ideal factors associated with the measurements, respectively.^20^
3
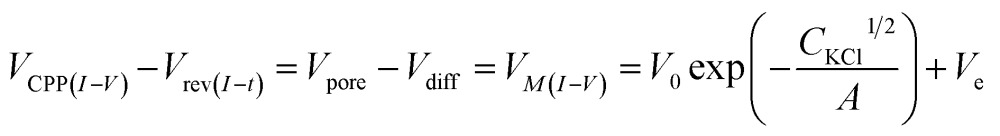



A correlation of *I*–*V* and *I*–*t* measurements by eqn (3) will reveal nanointerface parameters such as the surface potential (*V*_0_). The subtraction of *V*_redox_ from both types of measurements reveals the potential drop at the nanotip region (bulk-to-tip across the quartz membrane). At each tip concentration, *V*_M_ from symmetric tip–bulk concentrations equals that by the elimination of the diffusion potential in asymmetric concentrations (*i.e.* at 5 mM tip conc., 108 mV – 63 mV = 45 mV at 5 : 1 ratio and equals the 50 mV at 5 : 5 ratio within ±5 mV). The analysis suggests that the tip concentration plays a more significant role in transport measurements (data listed in Table S2†). The linear correlation in Fig. 5 strongly supports the correlation of eqn (3). The scattering at different bulk concentrations is attributed to the variation of local concentration gradients in the different measurements. The concentration range in this study is adopted to ensure stable and reproducible concentration gradients. With a predefined salinity and salinity gradient, this analysis offers the full picture of the dynamic ion transport across an asymmetric nanopore influenced by diffusion and migration.

### Implications on selective ion transport and history-dependent energy conversion at nanointerfaces

Three key factors have been correlated in this time-dependent ion transport analysis: the applied electric field, the surface electric field, and the salinity gradient. Each cross-point has a unique balance of the three factors in the corresponding current–potential analysis.

#### Selective ion transport and ion pumping

The intrinsic ion selectivity (with no bias applied) of a negatively charged nanopore can be characterized by the aforementioned analysis of the cation transference number. At a bias more negative than the cross-point potential, a low conductivity zone is established. The depletion of anion transport makes this zone highly cation selective due to negative surface charges. This is supported by a cation transference number approaching unity in earlier simulation and experimental studies.^37–39^ The high conductivity zone, at a bias more positive than the cross-point, is associated with high ionic strength and thus a decrease in ion selectivity.^37–39^ Therefore, the high conductivity region is more favorable for energy conversion applications (high power) rather than separation.

To return to the ion pump concept, the cross-points in Fig. 4 can be divided into three categories: *C*_tip_ = *C*_bulk_, *C*_tip_ < *C*_bulk_, and *C*_tip_ > *C*_bulk_. The transport of cations is considered because it constitutes the majority of the measured current signal. For *C*_tip_ < *C*_bulk_, the cations are pumped against their concentration gradient at the low conductivity side, driven by the applied potential but inhibited by the surface potential. On the high conductivity side, the cations move along the concentration gradient, and thus no pumping effect is expected. For *C*_tip_ > *C*_bulk_, the cations are pumped against their concentration gradient at the high conductivity side, driven by the applied potential and facilitated by the surface potential. At the low conductivity side, no pumping effect is expected. Four representative scenarios illustrate the physical picture of the corresponding transport processes (Fig. S9†). For applications that require anion selectivity, positively charged surfaces can be created by chemical modification, to enable favorable transport of anions over cations. The same principles apply to different salinity gradients.

#### History-dependent energy conversion at nanointerfaces

##### Cross-point signature

At the cross-point, a steady-state ion flux is established by the three factors: the current and potential at the cross-point indicate the external power/energy input required to balance the surface electrical field effects, plus the salinity gradient if adopted. Thus, the product of *I* × *V* at the cross-point represents the natural capacity of the employed nanointerface for the energy conversion process. The *I*(*t*) × *V*(*t*) at the cross-point is referred as power (*P*_cpp_). To evaluate the energy conversion or power in conventional electronic circuits, the open-circuit potential (OCP) and short-circuit current (SCC) are routinely analyzed because the triangular area in the *I*–*V* plots represents the maximum power (for a simple resistor load, *P* = *I* × *V* = *I* ^2^ × *R* = *V* ^2^/*R*). Similar analysis has been adopted to evaluate the maximum power of various nanochannels under a salinity gradient.^4,22,24^ It is important to realize that *P* = *I* × *V* = 0 in either OCP or SCC conditions (*I*_*V*=0_ × *V*_*I*=0_). Therefore, caution should be taken in nanopore systems because their resistance and capacitance vary and depend on the transport dynamics. Therefore, we propose *P*_cpp_ as a parameter with more rigorously defined physical meaning in the evaluation of transport related energy conversion at nanointerfaces, especially in systems with prominent rectification and hysteresis.

##### Power analysis and comparison

Importantly, *P*_cpp_ values are comparable in magnitude with the products of *I*_*V*=0_ × *V*_*I*=0_ for different nanopipettes, and comparable in general with those in related literature studies for power generation under salinity gradients (Table S3†).^40^ Unlike those literature studies performed at high pH conditions to induce higher surface effects, our measurements were performed at ambient pH to avoid the introduction of different types of ions that complicate the mechanism elucidation. Therefore, we expect further enhancement in the already-impressive power of our system at higher pH and/or high salinity gradient conditions.

##### Hysteresis in the charging–discharging kinetics

**Fig. 6:**
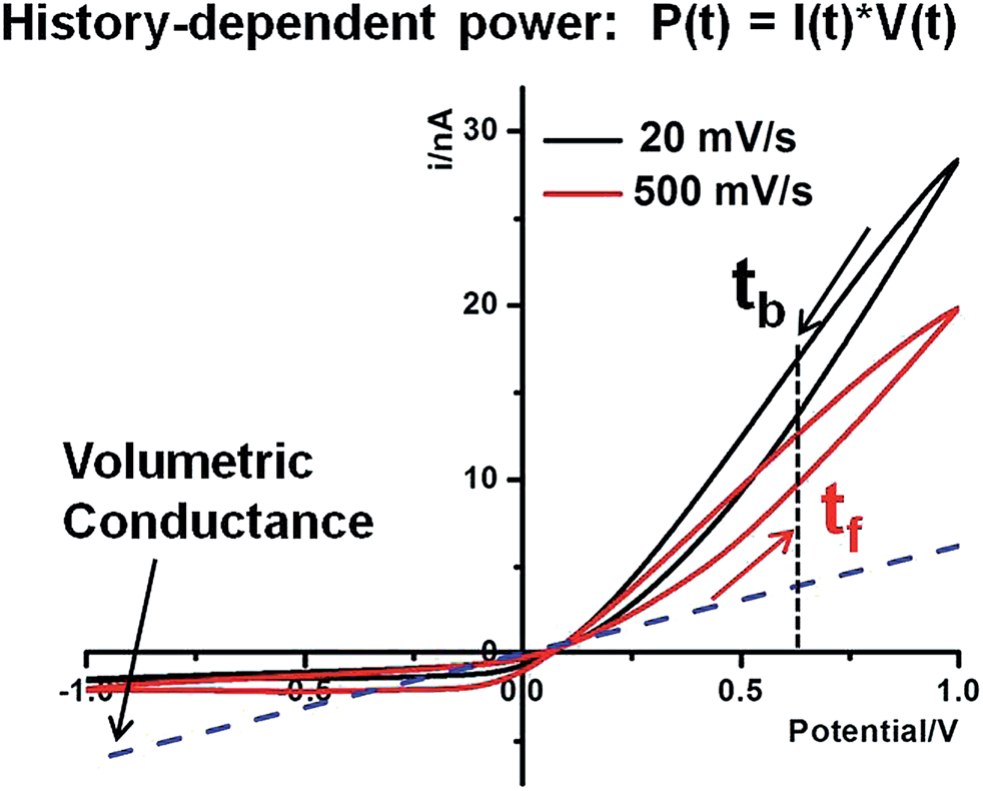
History-dependent power generation of the 60 nm nanopipette in 25 : 10 tip–bulk concentrations at 20 mV s^–1^ and 500 mV s^–1^. The volumetric conductance is estimated from tip concentration volumetric ohmic behaviors (blue dash line). The arrows near the current curves indicate the direction of the sweeping bias. Forward and backward scans are defined as away from and toward the cross-point, respectively.

History-dependent energy conversion is illustrated in Fig. 6. The following discussion is focused on high conductivity states that are better suited for energy applications. In reference to the volumetric conductance, drastic surface effects can be quantified at different time or bias domains. For example, at 0.60 V, *I*(*t*_b_) is larger than *I*(*t*_f_) due to the longer period during which larger surface effects accumulate (with a sustained contribution by *E*_s_). Similarly, both forward and backward currents are lower at higher scan rates due to less surface enhancement over shorter times at the same potentials. This surface enhancement functions regardless of salinity gradients. Given that the *P*_cpp_ describes the intrinsic capacity of individual nanostructures for power generation under a salinity gradient and establishes the physical meaning of such power generation under an external bias, further enhancement by the nanointerface in the power generation is anticipated if a positive bias were applied. The concept is analogous to the gain effect in field effect transistors. Furthermore, the forward and backward scans correspond to charging and discharging of the nano-supercapacitor, respectively. The hysteresis therefore suggests a delay in the charging (forward scan) as well as discharging (backward scan) driven by the external potentiostat. In other words, asymmetric nanopore structures and local surface electric field effects will alter the kinetics of the energy conversion. Our analysis suggests that, theoretically, the time constants of the energy conversion could be tuned by engineering nanointerfaces to achieve a desired power input–output for specific energy storage, power generation and other related applications.

## Conclusions

To summarize, time-dependent current potential features of individual conical nanopipettes are studied through which a fundamental understanding of the dynamic ion transport at nanoscale interfaces is established. By employing a salinity gradient across a conical quartz nanopipette, the hysteresis in the ion transport current with respect to a stimulating alternating electrical field is characterized by a non-zero *I*–*V* point and pinched *I*–*V* loops. The product of *I*–*V*, *i.e.* the power at the unique cross-point, is shown to be a more accurate and meaningful signature to characterize the dynamic ion transport or energy conversion at individual nanodevices. The hysteresis effect in *I*–*V* measurements has significant implications on the charging–discharging kinetics of energy devices such as supercapacitors. Furthermore, the conversion of the salinity gradient into electrical energy, or *vice versa*, on an asymmetric conical nanopore platform is demonstrated to be drastically enhanced by an intrinsic surface electrical field. The prerequisites to observe such time-dependent transport features and possible routes for further optimization are the asymmetry, such as the nanogeometry and fixed surface charges, that the ions experience within the time frame of stimulus or measurements. These findings suggest exciting opportunities to advance energy, separation and other related applications using broadly-defined channel-type nanodevices. The theoretical foundation and analysis methodology are universal and can be generalized to other structurally-defined nanoporous materials and nanodevices, and thus are believed applicable in broad energy and separation applications.

## Methods and materials

Quartz nanopipettes were fabricated with a P-2000 puller (Sutter Instrument Co.) using quartz capillaries (O.D.: 1.0 mm, I.D.: 0.7 mm). The pulling parameters of the nanopipettes are as follows: heat: 700, filament: 4, velocity: 60, del: 150, pull: 120. The experiments were performed with a Gamry Reference 600 (Gamry Co.). Two Ag/AgCl wires were used as the electrodes. One was immersed in the nanopipette as the reference electrode while the other was immersed in the bulk solution as the working electrode. The bias is therefore defined as outside *versus* inside. The current was recorded under an applied triangular potential waveform at 100 mV s^–1^ scan rate. The reported stable *I*–*V* curves were recorded after discarding the first few *I*–*V* segments and were confirmed by the overlap of the last five scans. To ensure the designed concentration gradient, the nanopipette was centrifuged for *ca.* 20 min after replacing the tip solution. Reproducible *I*–*V* responses in the symmetric setups were recorded between each asymmetric study to affirm that the proper concentrations were used. After each set of *I*–*V* measurements with the same tip concentration, additional *I*–*V* measurements under at least one asymmetric condition were conducted to demonstrate the stability and reproducibility of the measurements.
