# Supplementary material for: History-dependent ion transport through conical nanopipettes and the implications in energy conversion dynamics at nanoscale interfaces
Source: Chem Sci. 2014 Aug 20;6(1):588–95. doi: 10.1039/c4sc02195a (PMC5491961; doi:10.1039/c4sc02195a)
Supplement: Supplementary file 1 [file SC-006-C4SC02195A-s001.pdf]

Electronic Supplementary Material (ESI) for Chemical Science.
This journal is © The Royal Society of Chemistry 2014

Supporting Information For

History-dependent Ion Transport through Conical Nanopipettes and Implications in Energy Conversion Dynamics at Nanoscale Interfaces

Yan Li, Dengchao Wang, Maksim M. Kvetny, Warren Brown, Juan Liu and Gangli Wang

Department of Chemistry, Georgia State University, 50 Decatur St. SE, Atlanta, Georgia 30303

Corresponding author:
To whom correspondence may be addressed:
Dr. Gangli Wang, E-mail: gwang@gsu.edu Phone: 404-413-5507

Contents

Table S1 The current data at +/- 1.0 V of each curve in **Fig.3** are listed.

Table S2 Analysis of the cross point potential (V_{CPP}), measured zero-current potential (reversal potential V_{rev}) and calculated surface effective potential (V_M).

Table S3 Comparison of power generation from different nanopipettes with related literature under comparable experimental conditions.

Characterization of nanopipette geometry by imaging and conductivity analysis

Fig. S1 Side and top-view images of pulled nanopipettes by scanning electron microscopy.

Fig. S2 Overlaid i-V curves of the 60-nm nanopipette with symmetric 25:25 tip-to-bulk concentrations at different scan rates.

Fig. S3 Overlaid i-V curves of the 60-nm nanopipette in **a**) 1:1 **b**) 25:25 **c**) 1:25 **d**) 25:1 tip:bulk concentrations.

Fig. S4 Overlaid i-V curves of the 60-nm nanopipette in different tip:bulk concentration combinations at 100 mV/s and 500 mV/s scan rates.

Fig. S5 The overlaid i-V curves of four different sized nanopipettes in asymmetric tip:bulk concentration combinations.

Fig. S6 Analysis of cross point positions of a) 40 nm and b) 60 nm-radius nanopipette in different tip:bulk concentrations.

Fig. S7 Chronopotentiometry measurement of the 60 nm nanopipette with the current clamped at zero.

Fig. S8 Linear correlation of measured reversal potential V_{rev} with calculated redox potential V_{redox} from different nanopipettes.

Fig. S9 Illustration of the ion transport governed by three key factors.

References

Table S1 The current data at +/- 1V of each curve in **Fig. 3** are listed in Table. The same color code represents the same combination.

C_{tip}/mM	C_{bulk}/mM	i_{+1V}/nA	i_{-1V}/nA	C_{bulk}/mM	C_{tip}/mM	i_{+1V}/nA	i_{-1V}/nA
1	1	2.9	-0.4	1	1	2.9	-0.4
	5	5.4	-0.6		5	4.2	-0.6
	10	6.3	-0.7		10	6.0	-0.8
	25	7.8	-0.9		25	8.0	-1.2
25	1	8.0	-1.2	25	1	7.8	-0.9
	5	16.2	-1.4		5	15.5	-1.3
	10	23.8	-1.7		10	23.5	-1.7
	25	29.9	-2.1		25	29.9	-2.1

Table S2 Analysis of the cross point potential (V_{CPP}), measured zero-current potential (reversal potential V_{rev}) and calculated surface effective potential (V_M). The $V_M = V_{CPP} - V_{rev}$. At each tip concentration, the error is within 5 mV range at different bulk concentrations in general.

**C_{tip}/mM**	**C_{bulk}/mM**	**V_{CPP}/mV**	**V_{rev}/mV**	**V_M/mV**
1	1	59	5	54
	5	3	-52	55
	10	-17	-70	53
5	1	108	63	45
	5	51	1	50
	10	21	-22	43
10	1	129	88	41
	5	79	32	46
	10	49	4	45
25	5	95	63	32
	10	65	33	32
	25	31	0	31

Table S3 Comparison of power generation from different nanopipettes with related literature under comparable experimental conditions.

**Our Results**	**Nanodevice platform**	C_{tip}(mM):C_{bulk} (mM)	Power Estimation/pW				
			$P_{cpp} = V_{cpp} \times I_{cpp}$	$P = V_{i=0} \times I_{V=0}$	$P(t) = V(t) \times I(t)$		$P_{surface}$
1 (data reported in manuscript)	60-nm nanopipette	10:1	18.8	4.0	F(0.6 V)	2064	1200
					B(0.6 V)	2250	1400
2	60-nm nanopipette	10:1	7.0	3.2	F(0.6 V)	786	-67
					B(0.6 V)	930	77
3	40-nm nanopipette	10:1	12.2	8.4	F(0.6 V)	594	25
					B(0.6 V)	720	151
4	30-nm nanopipette	10:1	4.5	1.7	F(0.6 V)	1032	463
					B(0.6 V)	1092	523
**Literature**							
5	41-nm polyimide conical nanopore[1]	10:1 pH 10.5	N/A	10.9	N/A		
6	40-nm Boron-Nitride nanotude[2]	10:1 pH 11	N/A	15.2	N/A		
7	Less than 10 nm polymer (COO-terminated) nanopore[3]	100:10	N/A	15~20	N/A		

Here, P_{CPP} represents the cross point power generation. $P_{CPP} = V_{CPP} \times I_{CPP}$. P(t) represents the total transient power at a specific time. $P(t) = V(t) \times I(t)$.

VC refers to volumetric contributions (ohmic conductance by nanogeometry and solution resistivity). P_{VC} represents the power without contribution from surface charge, which is mainly determined by tip concentration. $P_{VC} = V(t)^2 \times G_{tip}$. G_{tip} is the tip solution conductance.

Note: the contribution from volume conductance to the overall conductance can be estimated from **Fig.3** in main text. Ignoring it in the above analysis of P_{VC} does not affect the general outcome of our analysis and has been confirmed by the analysis with square root tip*bulk.

$$G_{tip} = \frac{1}{R_{tip}} = \frac{1}{\rho_{tip}/\pi r \tan\theta + \rho_{tip}/4r} , \qquad P_{vc}(t) = V(t)^2 \times \frac{1}{\rho_{tip}/\pi r \tan\theta + \rho_{tip}/4r} .$$

$P_{surface}(t)$ is the power generation contributed from the surface at a specific time t. $P_{surface}(t) = P(t) - P_{VC}(t)$. Forward (F) and backward (B) refer to the bias V(t) being scanned away and toward the cross point respectively. Therefore, at the same bias, two current values are listed as the consequence of the hysteresis effects.

#1~#4: Cross point power generation: $P_{CPP} = V_{CPP} \times I_{CPP}$. V_{CPP} has been calibrated by substraction of electrode redox potential. For $P = V_{i=0} \times I_{V=0}$, here $V_{i=0} = V_{rev} - V_{redox}$. V_{rev} is obtained while holding current to be 0 A. $I_{V=0}$ is obtained while holding the potential to be 0 V. Original data of nanopipettes 2-4 were included in **Figs.S5-S6**. All data were collection under ambient pH. It is important to point out that the reported power in our system will be much higher at higher pH as reported in the references listed below.

#5: The data were copied directly from the literature Table.1 in ref. 1 with the same 10:1 asymmetric concentration. The solution pH was adjusted to 10.5 to increase surface charge effects, unlike ambient pH employed in other listed data.

#6: The current data $i_{V=0}$ and conductance data G were obtained directly from Supplementary Tab.I in ref. 2 with the same 10:1 asymmetric concentration. $V_{i=0} = I_{V=0}/G$ and was corrected from the electrode redox potential.

#7: The power data were read directly from Fig. 5 in ref. 3 while the asymmetric concentration is 100:10.

Characterization of nanopipette geometry

The radius and half cone angle θ of pulled quartz nanopipettes are characterized by scanning electron microscope (SEM) imaging shown in Fig.S1, and the conductivity analysis is described below.

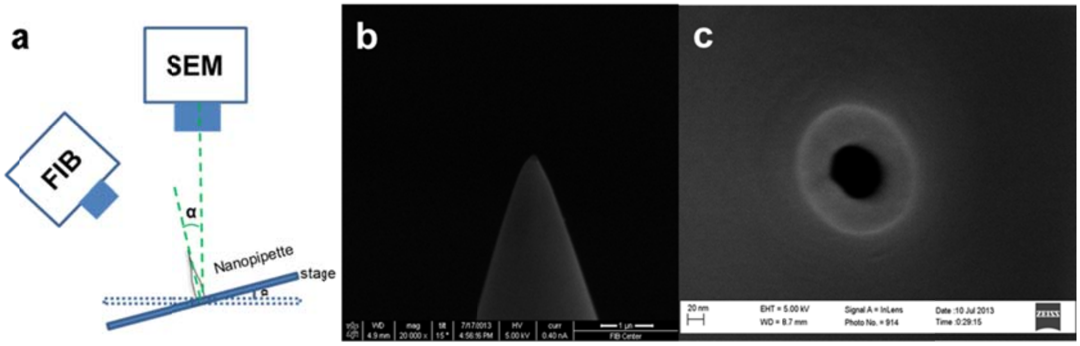

Fig.S1 a) Schematic diagram of a FEI Nova Nanolab 200 workstation with Focused Ion Beam (FIB) and SEM modes. The sample stage was tilted at 15° during imaging. **b**) Side-view of a nanopipette at 15° tilt angle under FIB/SEM mode. Scale bar is 1 μm. **c)** SEM top-view of the same nanopipette by Zeiss Ultra60 FE-SEM. The quartz nanopipette was spatter coated with a thin layer of Au/Pt alloy prior imaging. Scale bar is 20 nm.

The cone angle in **Fig.S1 b** is measured to be 37.5°. The half cone angle θ is determined to be 5.5° using equation: $\tan(2\theta) = \tan(37.5) \times \cos(90 - \alpha)$. Here, α is the tilt degree at 15°.

Conductivity analysis:

The size of the nanopipettes is normally calculated from conductivity results in literature. The nanopipette size/s used in those i-V studies were analyzed following the same procedure in literature detailed below.

The total pipette resistance comprises two major components: R_{geo} and R_{acc}, where R_{geo} is geometric resistance and R_{acc} is access resistance. R_{acc} is determined by its radius and solution conductivity given that the pipette orifice is disk-shaped in approximation.

$$R_{geo} = \frac{\rho h}{\pi r(r + h\tan\theta)},\ R_{acc} = \frac{\rho}{4r},\ R = R_{geo} + R_{acc} = \frac{\rho h}{\pi r(r + h\tan\theta)} + \frac{\rho}{4r}$$

Here, R is the resistance of solution. ρ is resistivity of the medium. h is the effective length. θ is the half cone. Because of the long stem of the pulled nanopipettes and the small radius employed in this study, r+htanθ is approximated to htanθ. Then,

$$R = \frac{\rho}{\pi r\tan\theta} + \frac{\rho}{4r},\qquad r = \frac{4\rho + \rho\pi\tan\theta}{4\pi R\tan\theta}$$

Here, θ is equal to 5.5^{o} and R can be calculated from the slope of i-V curves in high electrolyte concentration near zero bias that normally displays linear responses.

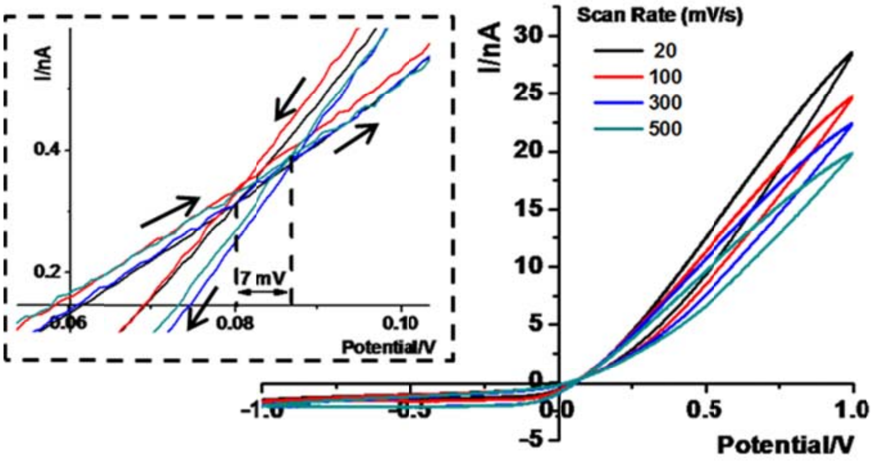

Fig. S2 Overlaid i-V curves of the 60-nm nanopipette in symmetric 25:25 tip:bulk concentrations at different scan rates. The inset shows the zoom in around the cross point. The largest difference in cross point potential is around 7 mV. The arrows show scan direction.

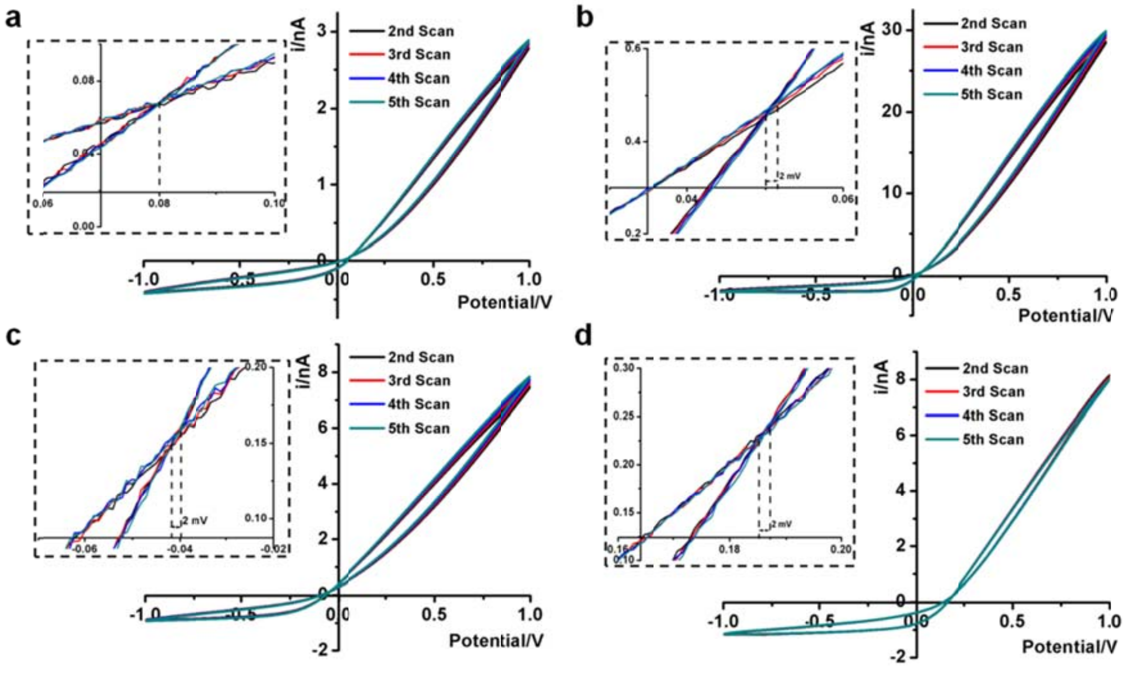

Fig. S3 Overlaid i-V curves of the 60-nm nanopipette in **a)** 1:1 **b)** 25:25 **c)** 1:25 **d)** 25:1 tip:bulk concentrations. Four complete scans at 100 mV/s scan rates are included in each panel. The first segment was discarded because the signals are affected by unknown pre-existing solution conditions. The insets show the zoom in part around the cross point potential.

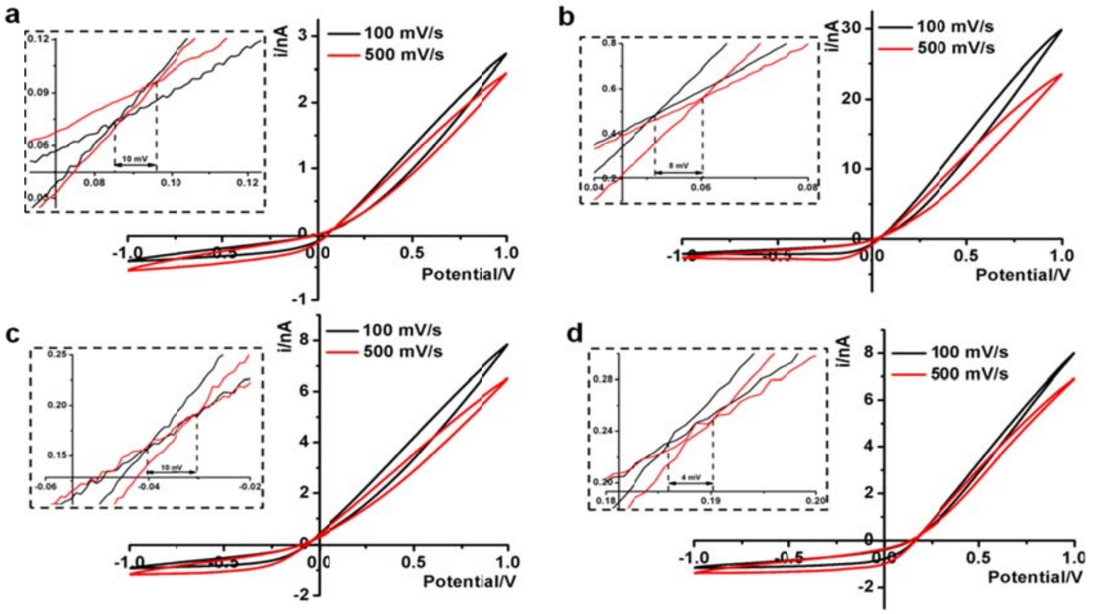

Fig. S4 Overlaid i-V curves of the 60-nm nanopipette in different tip:bulk concentration combinations at 100 mV/s and 500 mV/s scan rates. **a)** 1:1 **b)** 25:25 **c)** 1:25 **d)** 25:1. The insets show the zoom in around the cross point. The largest difference in cross point potential is around 10 mV.

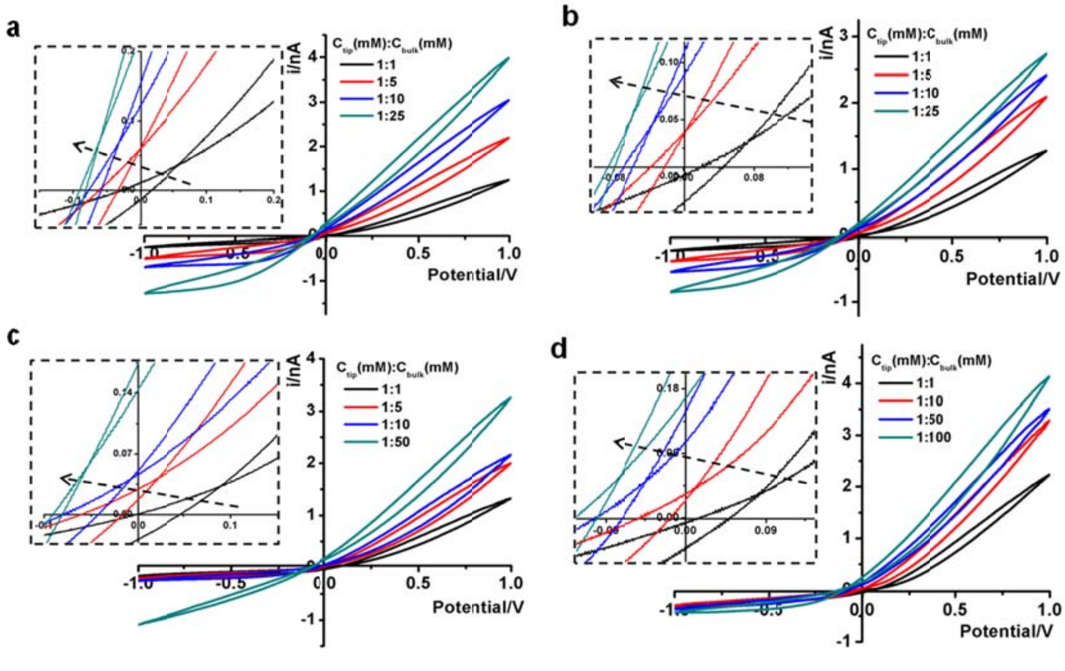

Fig. S5 The overlaid i-V curves of four different sized nanopipettes in asymmetric tip:bulk concentration combinations. **a)** 60 nm **b)** 80 nm **c)** 60 nm **d)** 30 nm-radius.

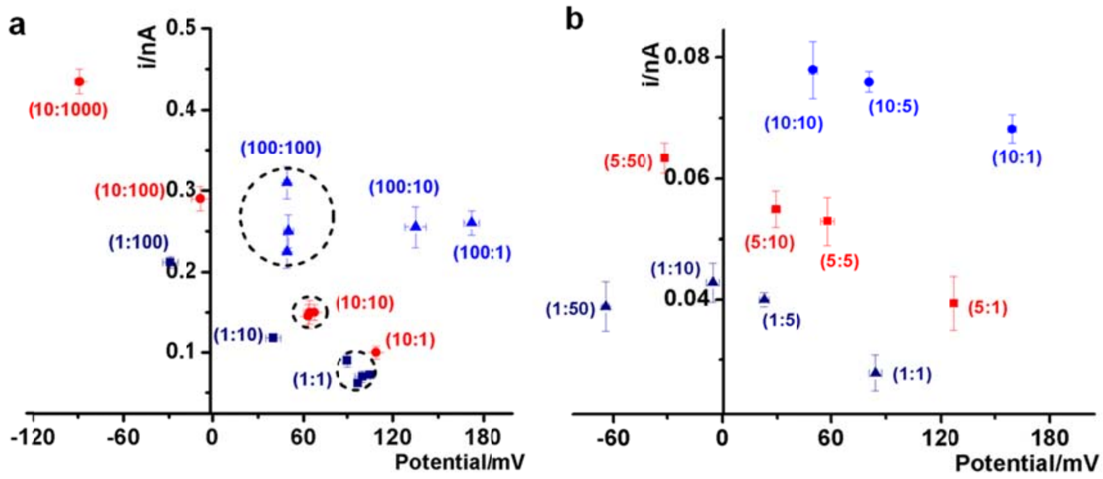

Fig. S6 Analysis of cross point positions of **a)** 40 nm and **b)** 60 nm-radius nanopipette in different tip:bulk concentrations. Redox potential has not been corrected. Note the (10:1000) data in panel a) follows the trend while the (1:50) data in panel b) deviate from the general trend. Both data points have greater concentration gradients then those employed in the main analysis.

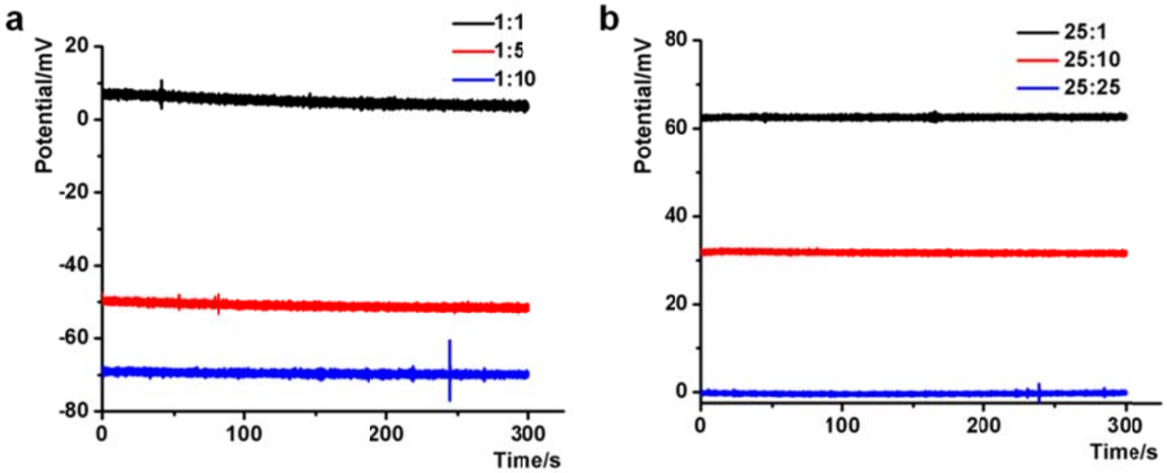

Fig. S7 Chronopotentiometry measurement of the 60 nm nanopipette with **a)** 1 mM and **b)** 25 mM KCl tip concentration with different bulk concentration that show stable potential-time traces.

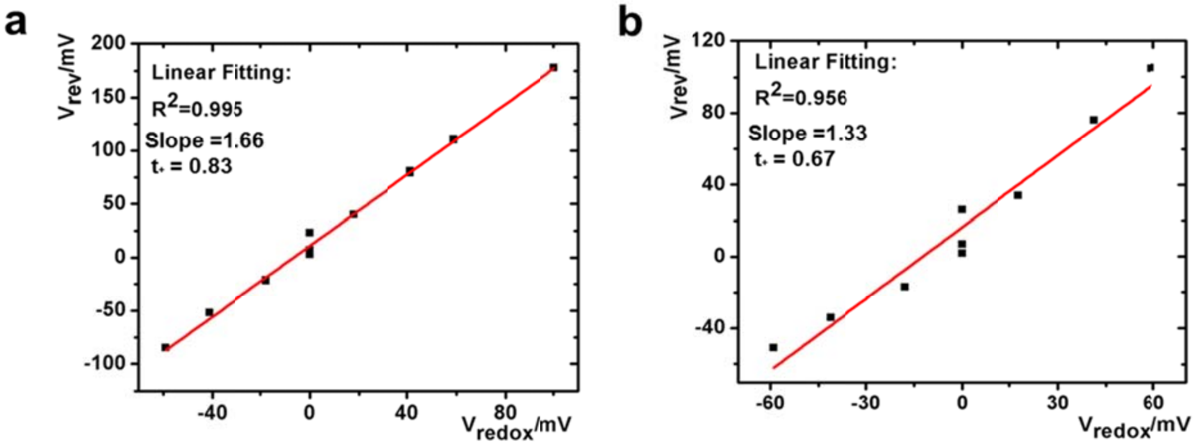

Fig. S8 Linear correlation of measured reversal potential V_{rev} with calculated redox potential V_{redox} from two different nanopipettes. **a)** 40-nm radius **b)** 60-nm radius.

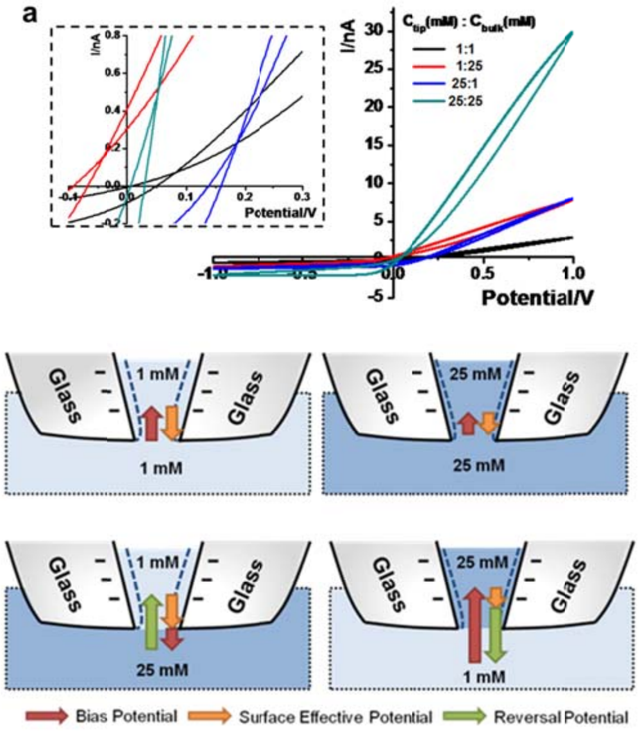

Fig. S9 Illustration of the ion transport governed by the three key factors. **a**) I-V curves of the 60-nm nanopipette in four respresentative concentration combinations. Enlarged view was shown in the inset. **b**) Four representative schemes of nanopipette with asymmetric 1:1, 1:25, 25:1 and 25:25 tip-to-bulk concentration gradients were shown. The dashed lines around glass surface show the thickness of double layer. The arrows were drawn based on real experimental value.

With symmetric tip-to-bulk setups, no diffusion potential is established. At cross point potential, the applied electric field balances the surface field effects, which is larger in 1:1 than that in 25:25 mM due to less effective electrostatic screening. After the introduction of an additional concentration gradient across the nanopipette, the applied potential needs to balance both surface potential and diffusion potentials at the cross point. At 25:1, even though the transmembrane potential is lower because the tip concentration is higher (compared to 1 mM inside), the concentration gradient and surface electric field have cumulative impacts on the ion flux at the transport limiting region inside the nanopipette. A bias magnitude equal to the sum of diffusion potential and surface potential is required to establish the cross point. Therefore, the cross point shifts toward more negative polarity if $C_{tip} < C_{bulk}$ and shifts toward more positive polarity if $C_{tip} > C_{bulk}$. Similar rationale can be applied to other concentration combinations or the transport of cations as main charge carriers and anions separately.

References:

1. W. Guo, L. X. Cao, J. C. Xia, F. Q. Nie, W. Ma, J. M. Xue, Y. L. Song, D. B. Zhu, Y. G. Wang and L. Jiang, *Adv. Funct. Mater.*, 2010, **20**, 1339.
2. A. Siria, P. Poncharal, A. L. Biance, R. Fulcrand, X. Blase, S. T. Purcell and L. Bocquet, *Nature*, 2013, **494**, 455.
3. J. Cervera, P. Ramirez, S. Mafe and P. Stroeve, *Electrochim. Acta.*, 2011, **56**, 4504.
